# Supplementary material for: BUB-1 and CENP-C recruit PLK-1 to control chromosome alignment and segregation during meiosis I in C. elegans oocytes
Source: eLife. 2023 Apr 17;12:e84057. doi: 10.7554/eLife.84057 (PMC10156168; doi:10.7554/eLife.84057)
Supplement: Figure 4—source data 1. — The two tables show the data obtained to generate the graph presented in Figure 4E, as total meiosis analysed (left) and the incidence of each phenotype (right). [file elife-84057-fig4-data1.zip › FIGURE 4-Source data.docx]

|  | **total n per meiosis stage** | | |  | **incidence of each phenotype per stage** | | | | |
| --- | --- | --- | --- | --- | --- | --- | --- | --- | --- |
|  |  |  |  |  |  |  |  |  |  |
| **Figure 4E** | **Metaphase** | **Anaphase** | **PBE** |  | **Metaphase** | | **Anaphase** | | **PBE** |
|  |  |  |  |  | **Mild** | **Severe** | **Mild** | **Severe** |  |
| **wild type** | 15 | 17 | 6 |  | 0 | 0 | 0 | 0 | 0 |
| ***bub-1(T527A)-/-*** | 37 | 46 | 22 |  | 13 | 10 | 11 | 1 | 1 |
| ***bub-1(T527A)+/-*** | 18 | 19 | 1 |  | 4 | 0 | 0 | 1 | 0 |
|  |  |  |  |  |  |  |  |  |  |
