# Supplementary material for: BUB-1 and CENP-C recruit PLK-1 to control chromosome alignment and segregation during meiosis I in C. elegans oocytes
Source: eLife. 2023 Apr 17;12:e84057. doi: 10.7554/eLife.84057 (PMC10156168; doi:10.7554/eLife.84057)
Supplement: Figure 8—source data 1. — The two tables show the data obtained to generate the graph presented in Figure 8C, as total meiosis analysed (left) and the incidence of each phenotype (right). [file elife-84057-fig8-data1.zip › FIGURE 8-figure supplement 2-Source data.docx]

|  | **total n per meiosis stage** | | |  | **incidence of each phenotype per stage** | | | | |
| --- | --- | --- | --- | --- | --- | --- | --- | --- | --- |
|  |  |  |  |  |  |  |  |  |  |
| **Figure 8-figure supplement 2C** | **Metaphase** | **Anaphase** | **PBE** |  | **Metaphase** | | **Anaphase** | | **PBE** |
|  |  |  |  |  | **Mild** | **Severe** | **Mild** | **Severe** |  |
| **wild type** | 12 | 12 | 4 |  | 0 | 0 | 2 | 0 | 0 |
| ***bub-1(RNAi)*** | 17 | 19 | 17 |  | 7 | 8 | 12 | 6 | 4 |
| ***hcp-4(T163A-/-*** | 10 | 12 | 9 |  | 0 | 0 | 2 | 0 | 0 |
| ***hcp-4(T163A)*-/- + *bub-1(RNAi)*** | 15 | 14 | 10 |  | 0 | 15 | 0 | 14 | 5 |
